# Incidence and predictors of virological failure among adult HIV patients on first-line antiretroviral therapy in Amhara regional referral hospitals; Ethiopia: a retrospective follow-up study

**DOI:** 10.1186/s12879-020-05177-2

**Published:** 2020-07-01

**Authors:** Chilot Desta Agegnehu, Mehari Woldemariam Merid, Melaku Kindie Yenit

**Affiliations:** 1 School of Nursing, College of Medicine and Health Sciences and Comprehensive Specialized Hospital, University of Gondar, Gondar, Ethiopia; 2 Department of Epidemiology and Biostatistics, Institute of Public Health, College of Medicine and Health Sciences, University of Gondar, Gondar, Ethiopia

**Keywords:** Virological failure, HIV, First-line antiretroviral therapy, Adult

## Abstract

**Background:**

Although the United Nations program on HIV/AIDS 90–90-90-targets recommends achieving 90% of viral suppression for patients on first-line antiretroviral therapy by 2020, virological failure is still high and it remains a global public health problem. Therefore, assessing the incidence and predictors of virological failure among adult HIV patients on first-line ART in Amhara regional referral hospitals, Ethiopia is vital to design appropriate prevention strategies for treatment failure and preventing the unnecessary switching to second-line regimens.

**Method:**

An institution-based retrospective follow-up study was conducted on 490 adult HIV patients. The simple random sampling technique was used, and data were entered into Epi data Version 4.2.0.0 and was exported to Stata version 14 for analysis. The proportional hazard assumption was checked, and the Weibull regression was fitted. Cox-Snell residual was used to test the goodness of fit, and the appropriate model was selected by AIC/BIC. Finally, an adjusted hazard ratio with a 95% CI was computed, and variables with *P*-value < 0.05 in the multivariable analysis were taken as significant predictors of virological failure.

**Results:**

The overall incidence rate of virological failure was 4.9 events per 1000 person-month observations (95%CI: 3.86–6.38). Users of CPT (AHR = 0.55, 95%CI: 0.31–0.97), poor adherence (AHR = 5.46, 95%CI: 3.07–9.74), CD4 Count <=200 cells/mm^3^ (AHR = 3.9, 95%CI: 1.07–13.9) and 201–350 cells/mm^3^ (AHR 4.1, 95%CI: 1.12–15) respectively, and NVP based first line drug regimen (AHR = 3.53, 95%CI: 1.73–7.21) were significantly associated with virological failure.

**Conclusion:**

The incidence rate of virological failure was high. CPT, poor adherence, low baseline CD4 count and NVP based first-line drug regimen were independent risk factors associated with virological failure. Therefore, strengthening HIV care intervention and addressing these significant predictors is highly recommended in the study setting.

## Background

Globally, it has been estimated that out of 36.9 million people living with HIV, 59% of them received Anti-retroviral Therapy (ART) [1]. Ethiopia is one of the sub- Saharan African countries most affected by the HIV epidemic and as estimated 610,335 people were living with HIV in 2018 [2]. A Highly Active Anti-retroviral Therapy (HAART) decreased HIV related morbidity and mortality associated with chronic HIV infection at a low cost of drug toxicity and increased patient survival [3, 4]. Nonetheless, the major challenge in ART treatment was reducing virological failure and increasing the occurrence of drug resistance, while most of the patients were experienced treatment failure [5]. Routine viral load monitoring can be carried out at 6 months on ART, at 12 monthsand then every 12 months thereafter if the patient is stable on ART to synchronizewith routine monitoring and evaluation reporting [6]. Virological failure, the most informative biomarker of treatment failure, [6, 7] has become a common public health problem among HIV patients on ART [6, 8]. For example, according to the World Health Organization (WHO) 2016 global report, 70% of patients experienced virological failure [9]. In sub- Saharan African countries, viral load suppression rate was 40.2–77.4% [10], and 24% of the adult patients on first-line ART experienced virological failure within 12 months of ART initiation [11]. In the Ethiopian public health facilities, virological failure was estimated to be 11.9% [12]. Ethiopia has adopted the UNAIDS 90–90-90 treatment target by 2020 [13], and other countries have planned to reach 90% viral suppression among all people receiving ART [5]. However, evidence indicated that more than 10% of patient plasma viral load was not suppressed after 6 months of the first-line ART treatment [14–17]. Studies indicated that there was a high incidence of virological failure. For example, in Tanzania, 14.9% [18], southeast Uganda 8.67 events per person-year follow up (PYFU) [19], and in india10.7 per 100 PYFU [20], and various factors were associated with virological failure. Accordingly, poor adherence [17, 20–26], lower CD4 count at baseline [17, 19, 25–31], age [17, 21, 25, 32], TB/HIV co-infection [28], and non-disclosure status [33] were associated with virological failure.

Identifying and handling the determinants of virological failure and reducing its incidence is used to realize the 90–90-90 treatment target and achieve sustainable development goal 3. To create an HIV free generation and stop HIV epidemic, early detection of virological failure on first-line ART patients is very important for better preservation of the efficacy of second-line regimens. Maintaining a low viral load is important for patients to prevent the progression of AIDS and associated co-infections; yet, there is only limited evidence on the incidence of virological failure and its predictors. Therefore, this study aimed to estimate the incidence of virological failure and identify its predictors among adult HIV patients on first-line ART in Amhara regional referral hospitals.

## Method

### Study design and setting

**Fig. 1:**
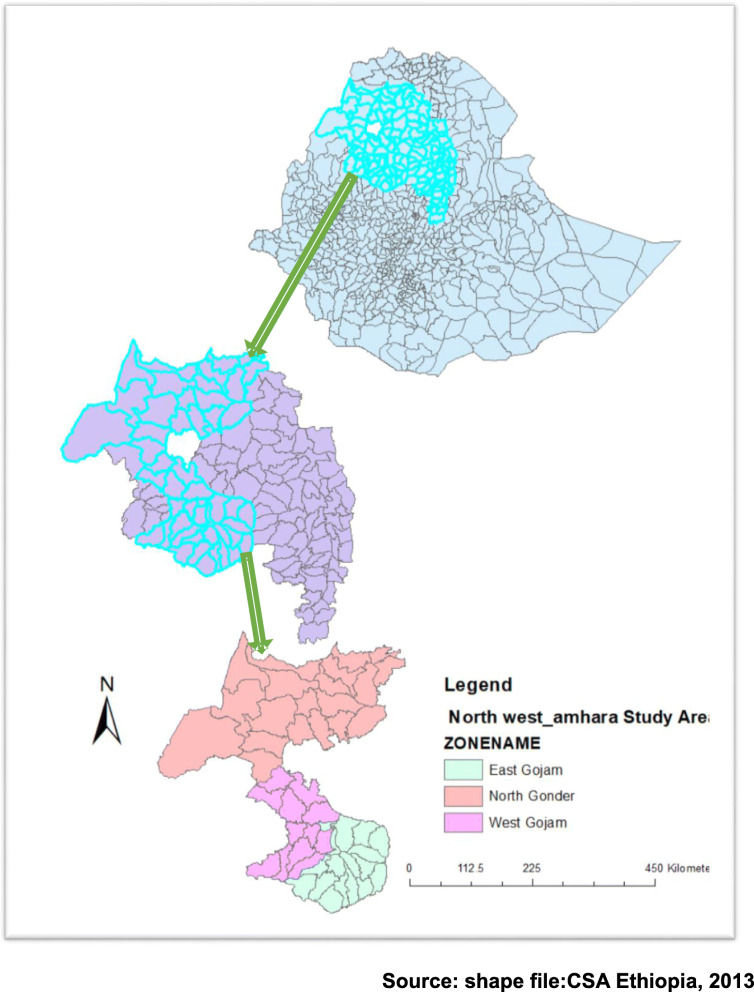
Map of Study area (northwest Amhara) that shows three zones which contain three referral hospitals

An institutional-based retrospective follows up study was conducted from September 2015 to December 2018 in three Amhara regional referral hospitals including; the University of Gondar comprehensive specialized hospital, Bahirdar Felegehiwot referral hospital and Deberemarkos referral hospital. As part of the national AIDS control Program, in Amhara regional referral hospitals have been providing free ART services from 2005 to date. The hospitals provide clinical care, including laboratory and pharmacy services (Fig. 1).

### Population and sample

The target population in this study were all adult HIV patients ≥15 years’ age on the first line ART r enrolled in ART clinic in the study period Amhara regional referral hospitals and the study population were patients enrolled in this referral hospitals ART clinic from September 2015 to April 2018. By using simple random sampling technique adult HIV patients on first-line ART treated for at least 9 months were included.

We used survival sample size calculation power approach using Stata 14.1 software with Cox proportional hazard assumptions. Sample size was calculated for the four predictor variables including Age 15–24 years (HR = 4.4), poor adherence (HR = 3.25), duration of ART (HR = 6.62) and ART regimen change (HR = 3.95) from retrospective follow up study done in Adama medical college [24] (Table 1). Accordingly,the minimum sample size was 513 by considering 10% incomplete data. Finally 490 patient charts fulfilled the inclusion criteria were included in the analysis.

**Table 1:** Sample size determination of adult HIV patients on first line ART in Amhara regional referral hospitals; Ethiopia from September 2015 to December 2018

Assumptions	Probability of event	Variables	Hazard ratio	Probability of withdrawal	Sample size
Power = 80	0.06	Age 15–24 years	4.4	0.1	265
Significance level(α) = 0.05	**0.049**	**Poor adherence**	**3.25**	**0.1**	**513**
Allocation ratio 1:1	0.09	Duration of ART	6.62	0.1	109
Two tailed	0.06	ART regimen change	3.95	0.1	309

### Variables of the study

The dependent variable was incidence of virological failure, whereas the independent variables were socio-demographic characteristics (Age, sex, residency, marital status, belonging to support group and HIV disclosure status), anti-retroviral medication related and Clinical characteristics (adherence, change of ART regimen, baseline BMI, base line WHO clinical stage, ART duration, CD4 count, TB/HIV co-infection, base line hemoglobin, past opportunistic infection and base line functional status).

Survival time was defined as time in month from the start of first line ART treatment to the development of virological failure. Event was defined as patients who developed virological failure during the follow up time. Virological failure is defined as during the follow up time viral load above 1000 copies/ml based on two consecutive viral load measurements in 3–6 months, with enhanced adherence support following the first viral load test [6]. Censored was defined when the study participants lost, transfer out, died and free from event during the follow up time.

Adherence to ART medications was classified as good, fair, and poor according to the percentage of drug dosage calculated from the total monthly dose of ART drugs as follows**:** Good (equal to or greater than 95% or ≤ 3 doses was missed per month), Fair (85–94% or 4–9 doses was missed per month), or Poor (less than 85%. Anemia**:** was classified for women < 12 g/dl were Anemic and ≥ 12 were not anemic whereas for men < 13 g/dl were anemic and ≥ 13 were not Anemic [18].

### Data collection procedures and quality control

Data were extracted by using the appropriate data extraction tool adapted from the national HIV intake and follow-up care form. Data were collected by six nurses working in the ART clinics and training on the objective of the study and they used structured data extraction tools from patient’s medical records, such as pre ART intake, follow up, and laboratory request forms. To ensure the quality of data, a one-day training was given to six data collectors and three supervisors on the significance, variables of the research and how to review t documents by using the extraction tool.

### Data analysis

Data was entered into EPI-Data version 4.2.0.0 and exported to Stata 14 software for recording and analysis. Descriptive statistics and Incidence rate (IR) was calculated for the events of virological failure. The Kaplan Meier (KM) failure curve and log-rank test were used to describe the survival experiences of categorical variables. The proportional hazard assumption was checked both graphically and using a Schoenfield residual test. The goodness of model fitness was also checked using the Cox-Snell residual plot.

The appropriate model for the data was selected based on the Akaike Information Criterion (AIC), Bayesian Information Criterion (BIC) and log-likelihood ratio (LL). Hazard Ratio (HR) was used as a measure of association. Parametric survival models were fitted by assuming baseline hazard distribution. The frailty model was taken into account by introducing the random effect model for time-to-event data, by adding a frailty term “H”. Thus, both univariate and shared frailty models were tested by considering different parametric distributions and the frailty distribution (gamma and inverse Gaussian). A more parsimonious model was chosen using BIC and AIC. The model with the smallest AIC was considered as an appropriate fitted model. Variables having *P*-values ≤0. 2 in the bi-variable analysis were entered into the multivariable analysis and variables with *P*-value ≤0. 05 and an adjusted hazard ratio (AHR) with a 95% confidence interval (CI) were considered as statistically significant predictors of virological failure.

## Results

### Description of study participants

**Fig. 2:**
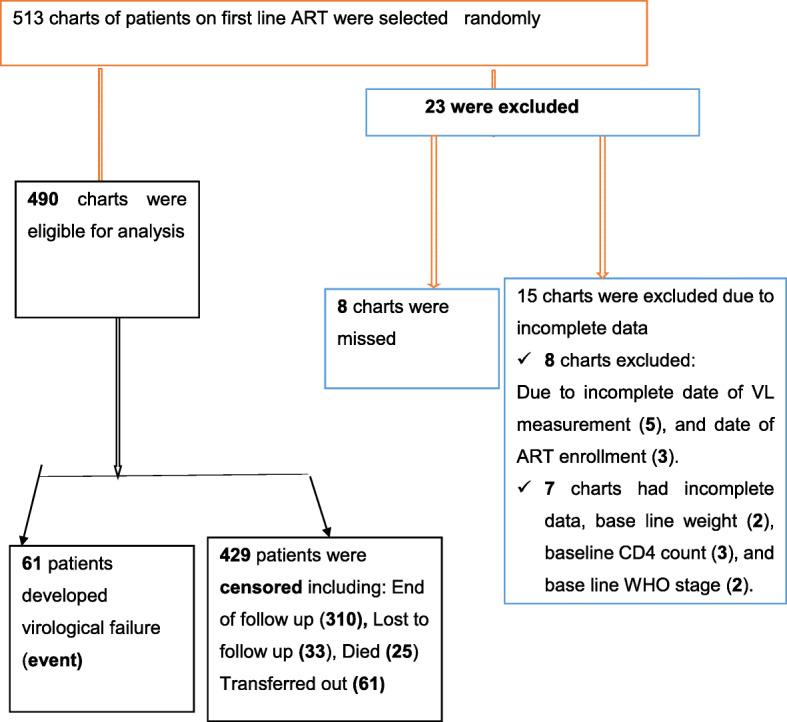
Flow chart showing a selection of adult HIV/AIDS patients on the first line ART in Amhara regional referral hospitals from September 2015 to December 201

About 2251 adult HIV patients on first-line ART were enrolled between September 2015 and December 2018 in northwest Amhara referral hospitals. Based on our sample size determination, 513 medical charts were included of which 23 medical charts were excluded due to missed charts and incomplete data. As a result a total of 490 patients were included in the analysis (Fig. 2).

### Socio-demographic characteristics

The median age of the patients was 32 with IQR [28, 34] years. More than half, 287 (58.57%) of the patients were female. Of all patients, 440(89.8%) were orthodox Christian. Three-fourths (76.94%) of the patients were urban dwellers and 195(39.8%) were married at the moment. Among the total, 296(60.41%) of the patients had disclosed their HIV status and 272(55.51%) were self-employed. More than three-fourth 393(80.2%) had support groups (caregivers) (Table 2).

**Table 2:** Baseline socio-demographic characteristics of adult HIV patients on first-line ART in Amhara regional referral hospitals, Ethiopia from September 2015 to December 2018 (*N* = 490**)**

Variables	Frequency	Percent
Age in year		
15–24	78	16
25–34	196	40
35–44	142	29
> =45	74	15
Sex		
Male	203	41.43
Female	287	58.57
Religion		
Orthodox Christian	440	89.8
Muslim	36	7.35
Others^a^	14	2.85
Occupation		
Unemployed	104	21.22
Employed	90	18.37
Daily laborer	24	4.90
Self-employed	272	55.51
Residency		
Urban	377	76.94
Rural	113	23.06
Educational status		
No education	128	26.12
Primary	122	24.9
Secondary and above	240	48.98
Marital status		
Single	129	26.33
Married	195	39.8
Divorced	124	25.31
Widowed	37	7.55
Separated	5	1.02
Disclosure status		
Disclosed	296	60.41
Not disclosed	194	39.59
Support group/caregiver		
Yes	393	80.2
No	97	19.8

^a^ Others protestant, catholic

### Baseline clinical and anti-retroviral medication-related characteristics

Of the total 490 patients, 271(55.31%) took Cotrimoxazole preventive therapy (CPT). The majority, 437(89.18%), of the patients were treated for first-line Efavirenz (EFV) based ARV drug regimen. Nearly two-thirds (62.04%) had baseline WHO clinical stage I/II and 414(84.49%) had good ART adherence status. Almost three –fourths 265(74.49%), took Isoniazid preventive therapy (INH); 398(81.22) had no TB/HIV co-infection; only 106(21.63%) were anemic, while 385(78.57%) could perform routine activities (Table 3).

**Table 3:** Baseline clinical and antiretroviral medication-related information among adult HIV patients on first-line ART in Amhara regional referral hospitals, Ethiopia from September 2015 to December 2018 (*N* = 490)

Variables	Frequency	Percent
Past opportunistic infection		
Yes	150	30.61
No	340	69.39
CPT		
Yes	271	55.31
No	219	44.69
INH		
Yes	125	25.51
No	265	74.49
TB/HIV co-infection		
Yes	92	18.78
No	398	81.22
Baseline functional status		
Working	385	78.57
Ambulatory/Bedridden	105	21.43
Adherence on ART		
Good	414	84.49
Fair/poor	76	15.51
First-line drug regimen		
EFV based	437	89.18
NVP based	53	10.82
Baseline hemoglobin level(g/dl)		
Anemic	106	21.63
Not anemic	384	78.37
Baseline clinical WHO stage		
Stage I/II	304	62.04
Stage III/IV	186	37.96
Body mass index		
Severely underweight	64	13.06
Moderate underweight	94	19.18
Normal	278	56.73
Overweight	54	11.02

### The incidence rate of virological failure

Four hundred ninety (490) adult HIV patients in the first-line ART were followed for different periods with a total of 12,281.53 person-months (PM) of observations. Patients were followed for a minimum of 8.9 and a maximum of 40.33 months; 61(12.4%) of patients developed virological failure during the follow-up period (95%CI: 9.7–15.6). Hence, the overall incidence rate of virological failure in this follows up was 4.9 (95%CI, 3.86–6.38) per 1000 PM of observations. The cumulative hazard of virological failure at 12, 24 and 36 months was03, 7.75 and 7.65 per 1000 PM observations, respectively.

The overall IR of virological failure at the University of Gondar comprehensive specialized hospital, Deberemarkos referral hospital, and Feleghiowt referral hospital was 5.63 (95%CI, 3.7–8.4), 5.3 (95%CI, 3.3–8.4) and 4.1 (95%CI, 2.6–6.4) cases per 1000 PM observations, respectively.

**Fig. 3:**
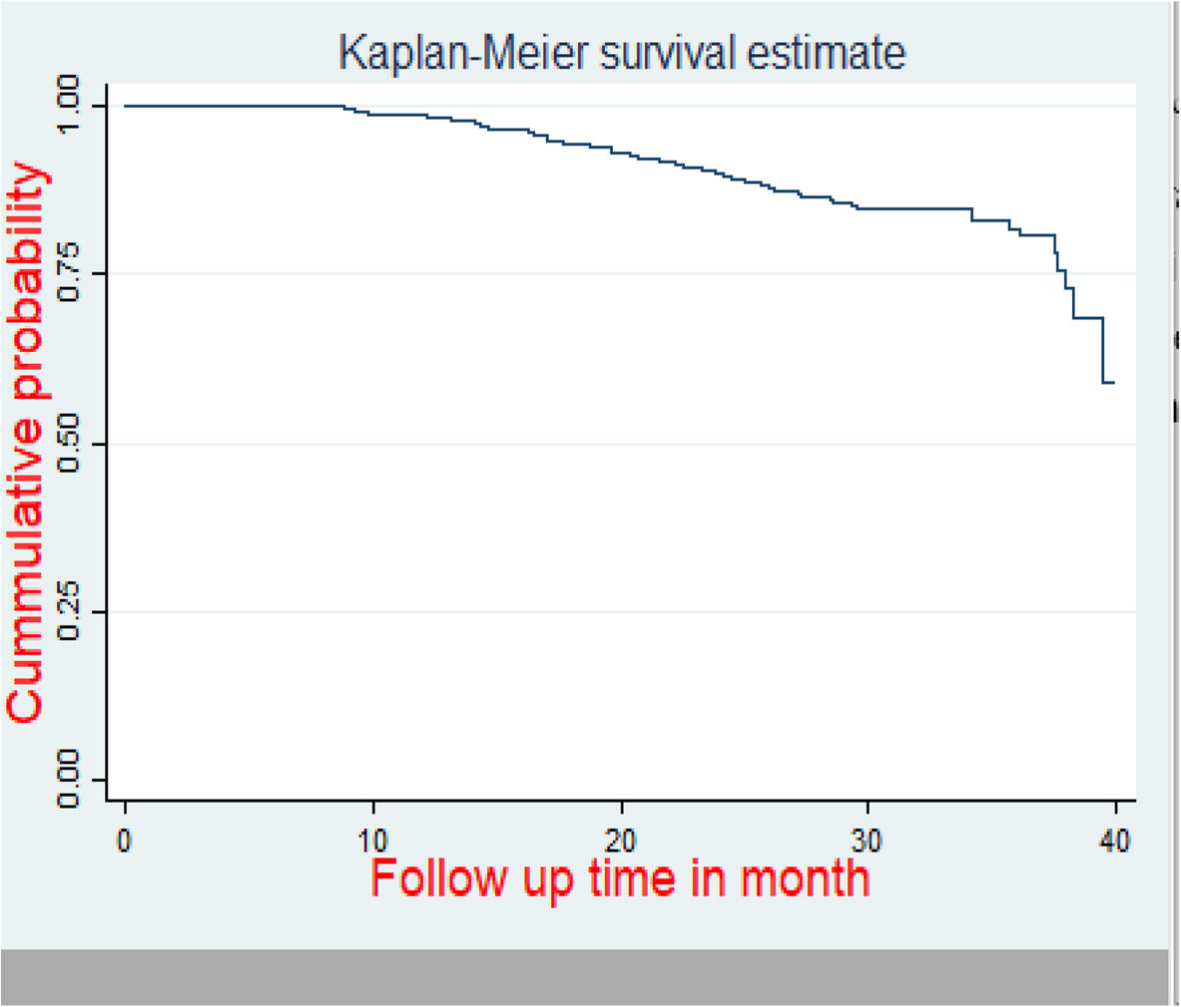
Kaplan Meier failure hazard function of virological failure of HIV/AIDS patients on first line ART in Amhara regional referral hospitals, Ethiopia from September 2015 to December 2018

A graph of the Kaplan Meier (KM) failure function was used to describe the cumulative IR of virological failure over the follow-up period. The cumulative probability of surviving or being free from the event of interest at the end of 10, 20, 30 s and 40 months was 98.9, 92, 80, and 59%, respectively (Fig. 3).

### Predictors of time to virological failure

The Kaplan Meier failure function and log-rank test were used to show differences in survival experiences among different groups of categorical variables at baseline.

**Fig. 4:**
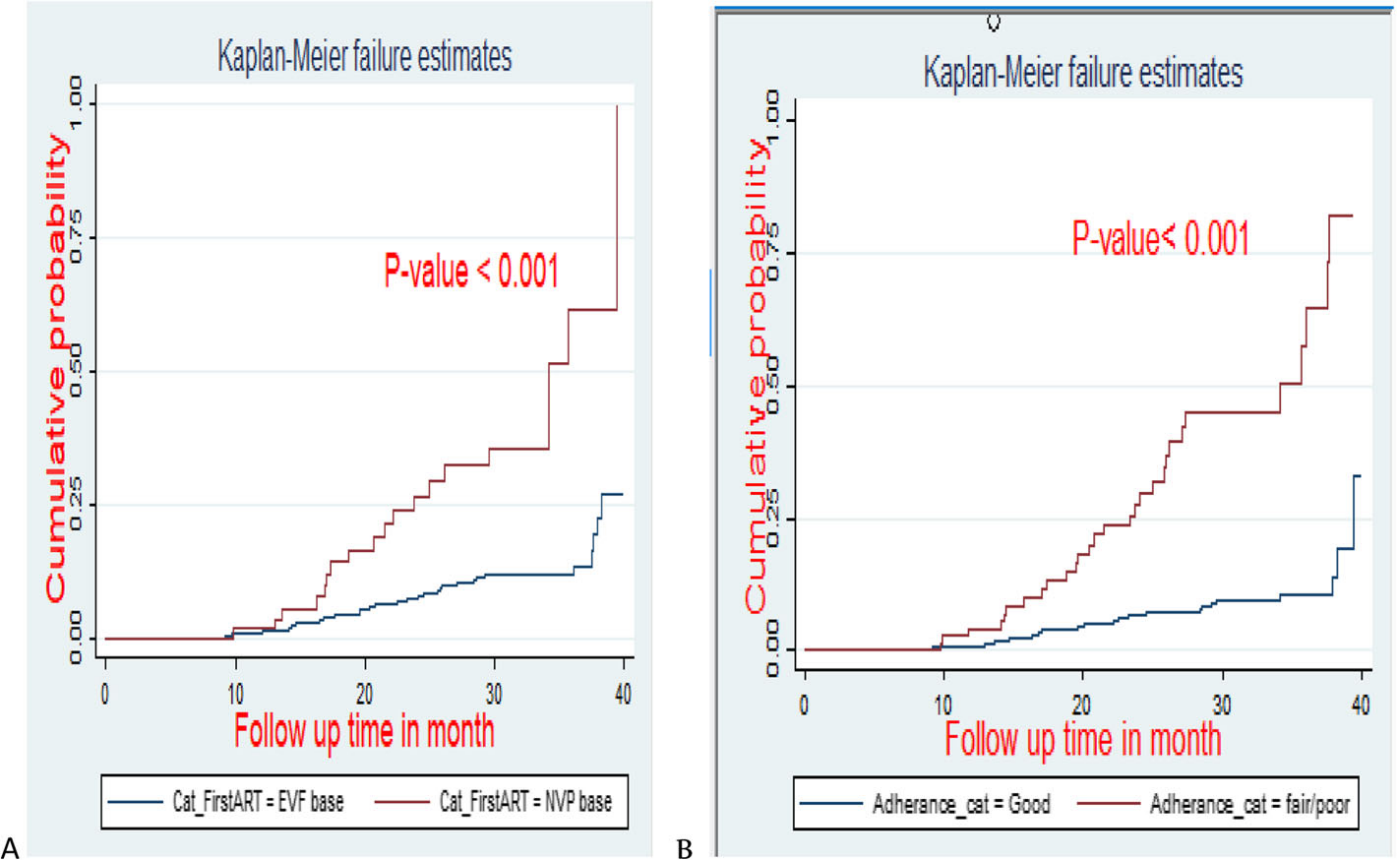
Kaplan Meier hazard curve by (A) first line drug regimen (B) adherence status of virological failure in adult HIV/AIDS patents on first line ART in Amhara regional referral hospitals; Ethiopia from September 2015 to December 2018. UoGCSh = university of Gondar Comprehensive specialized hospital. FHRH = Felege hiwot referral hospital. DMRH = Debremarkos referral hospital

In case of survival experience without adjusting other covariates, there were significant variations between EFV and NVP based regimen (*P* < 0.001) and in those who were in poor and Good adherence (*P* < 0.001) (Fig. 4).

**Fig. 5:**
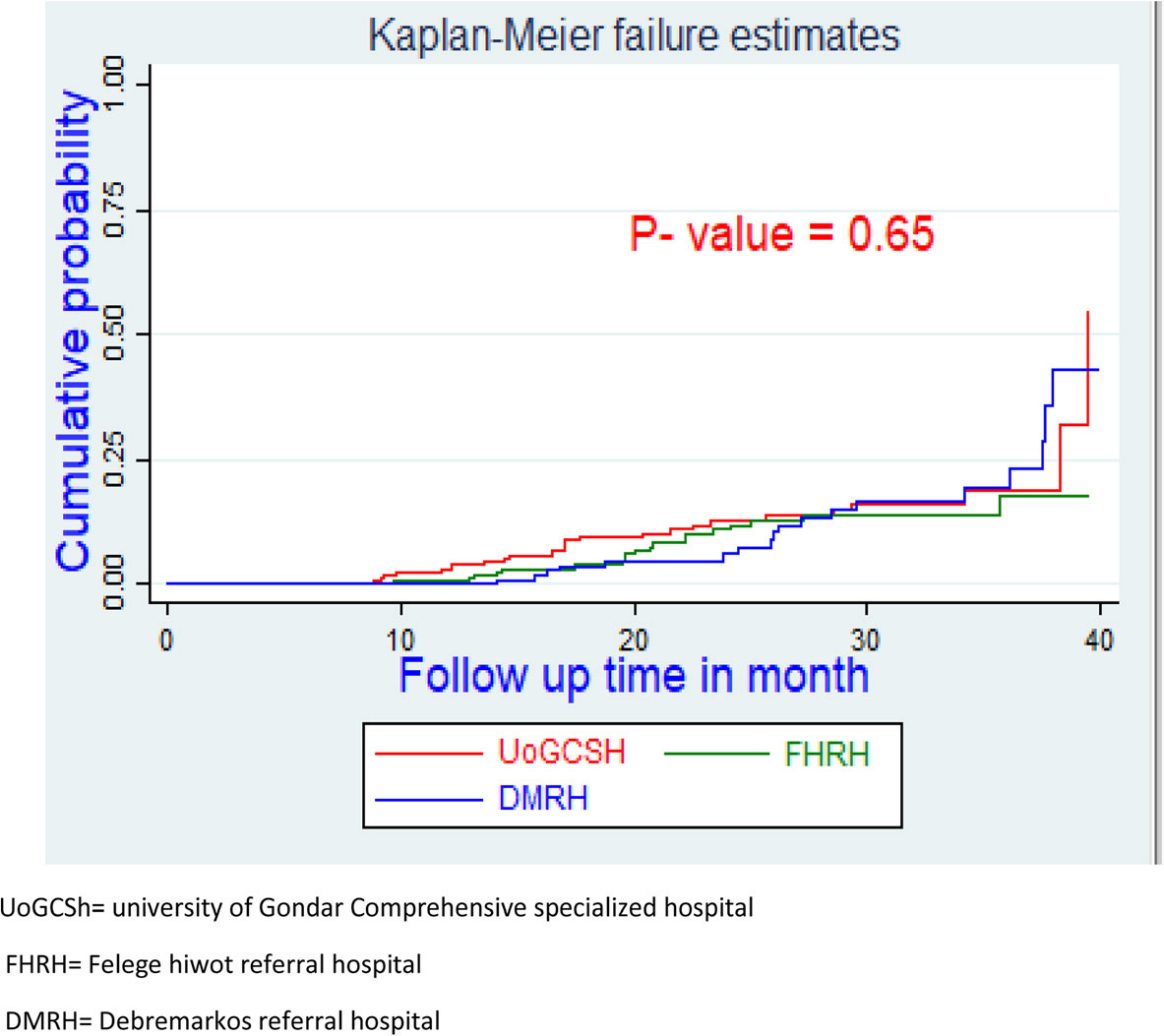
Kaplan Meier failure curve of by hospitals virological failure in adult HIV/AIDS patents on first line ART in Amhara regional referral hospitals; Ethiopia from September 2015 to December 2018

The survival curve plotted below indicated the estimated hazard curves of the hospital and the log-rank test used for checking the differences in hazard curves displayed. There was no overall difference between the hazard curves of the hospitals and supported by the log-rank test (Log-rank Chi-square [2] = 0.86, *p* = 0.65) (Fig. 5).

### Assessing the proportional hazard assumption

The proportional hazard assumption states that the risk of failure of the study subjects must be the same no matter how long they are followed. The global test of the proportional hazards assumption based on the Schoenfeld residuals was done, and it was found that all of the covariates and in the full model satisfied the proportional hazard assumption (Chi-square = 12.14, *p*-value = 0.52).

### Model comparison

After the proportional hazard assumption was checked, both semi-parametric and parametric proportional hazard models were fitted to estimate the survival time to virological failure and identify its predictors among HIV patients on first-line ART. Information criteria (AIC, BIC) and log-likelihood were used to select the most parsimonious models for the data set.

Based on this, the Weibull regression with the (**AIC = 313.15, BIC = 405.32)** model was more efficient than Cox proportional hazard and other parametric models.

On the other hand, frailty effect by treatment hospitals was not a statistically significant variance between individuals among hospitals and also among individuals (Table 4).

**Table 4:** Summary of model comparison among the Cox proportional hazard model, parametric Cox- Regression models and frailty models using AIC, BIC LR criteria

Model	Baseline Hazard	Frailty	Variance	AIC	BIC	Log-likelihood
Cox regression	Unspecific			622.50	706.38	− 291.25
Weibull regression	**Weibull**			**313.15**	**405.43**	− 134.57
Univariate frailty	Weibull	Gamma	1.4e^−07^(*p* = 1.00)	315.16	411.42	− 131.23
Univariate frailty	Weibull	Inv_Gaussian	2.81e^−07^(*p* = 1.00)	315.16	411.62	−134.57
Shared frailty (Hospital)	Weibull	Gamma	8.42e^−07^(*p* = 1.00)	315.16	411.62	.131.23
Exponential	Exponential			372.74	460.82	− 165.37
Gompertz	Gompertz			322.04	426.27	− 139.02
Loglogistic regression	Log logistic			318.81	411.09	− 137.40
Lognormal regression	Log normal			325.21	417.48	− 140.60

**Fig. 6:**
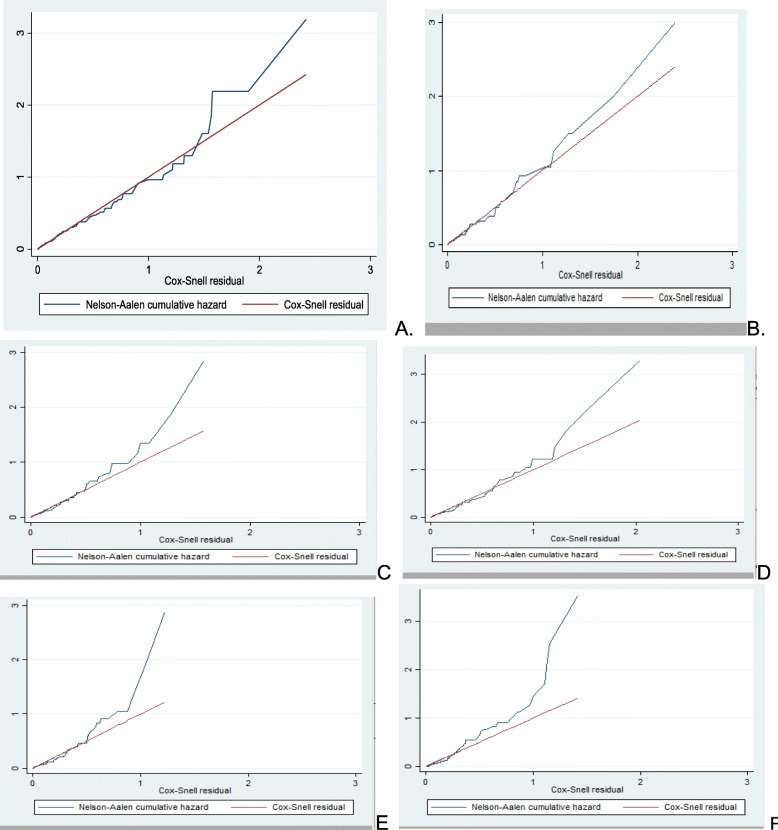
Plot of Nelsen-Aalen cumulative hazard function against Cox-Snell residual obtained by fitting Weibull (A), Gompertz(B), log logistic (C), Cox (D), lognormal (E)F(exponential) models for virological failure of HIV/AIDS patients on first line ART in Amhara regional referral hospitals; Ethiopia from September 2015 to December 2018

The Cox- Snell residuals versus Nelson-Aalen cumulative hazard function were obtained by fitting the Cox, Weibull, Gompertz, lognormal, log-logistic and exponential models to the data. It can be seen that the plot of the Nelson-Aalen cumulative hazard function against the Cox-Snell residuals has a linear pattern making a straight line through the origin of the Weibull model when compared to cox, Gompertz, lognormal, log-logistic and exponential models. This suggests that the Weibull regression model provided the appropriate fit for this data set (Fig. 6).

### Model diagnosis

The finding of the Bi-variable Weibull regression showed that age, sex, educational status, occupational status, CPT, baseline functional status, adherence, TB/HIV co-infection, first-line drug regimen, hemoglobin level, baseline CD4 count, baseline Clinical WHO stage and past opportunistic infection were significantly associated with virological failure.

However, in the multivariable analysis CPT, adherence, first-line drug regimen, and baseline CD4 count were statistically significant with virological failure (Table 5).

**Table 5:** Bi-variable and multi-variable Weibull regression analysis for predictors of virological failure among adult HIV patients on first-line ART in Amhara regional referral hospitals; Ethiopia from September 2015 to December 2018(*N* = 490)

Variables	categories	Status		CHR(95%CI)	AHR(95%CI)
		Event	Censored		
Age in year	15–24	13	65	2.02 (0.77–5.33)	0.67 (0.19–2.35)
	25–34	27	169	1.68 (0.69–4.07)	1.24 (0.47–3.23)
	35–44	15	127	1.36 (0.52–.3.50)	0.80 (0.29–2.21)
	> = 45	6	68	1	1
Sex	Male	30	173	1.48 (0.90–2.46)	1.46 (0.83–2.57)
	Female	31	256	1	1
Educational status	No education	15	113	1	1
	Primary	22	100	1.62 (0.84–3.12)	1.32 (0.64–2.71)
	Secondary above	24	216	0.81 (0.42–1.56)	0.71 (0.33–1.49)
Occupation	Un employed	19	85	2.21 (1.23–3.97)	1.52 (0.73–3.15)
	Employed	9	81	1.06 (0.50–2.25)	1.88 (0.80–4.42)
	Daily laborer	5	19	2.54 (0.99–6.58)	1.35 (0.49–3.75)
	Self-employed	28	244	1	1
CPT	Yes	32	239	1	1
	No	29	190	1.40 (0.84–2.32)	**1.8 (1.03–3.16**)*
Baseline functional status	Working	40	345	1	1
	Ambulator/bedridden	21	84	1.98 (1.16–3.36)	1.23 (0.65–2.32)
Adherence	Good	32	382	1	1
	Fair/poor	29	47	6.16 (3.7–10)	**5.46 (3.06–9.74**)*
TB/HIV-co-infection	Yes	15	77	1.91 (1.07–3.43)	1.27 (0.65–2.51)
	No	46	352	1	1
First-line drug regimen	EFV based	42	395	1	1
	NVP based	19	34	3.82 (2.22–6.5)	**3.53 (1.73–7.21**)*
Hemoglobin level	Anemic	25	81	2.6 (1.57–4.35)	1.46 (0.83–2.57)
	Not anemic	36	348	1	1
Baseline CD4 count	<=200	29	163	2.83 (0.86–9.3)	3.9 (1.07–13.9)*
	201–350	18	110	2.47 (0.72–8.41)	4.1 (1.12–15)*
	351–500	11	90	1.96 (0.54–7.04)	2.14 (0.54–8.5)
	> 500	3	66	1	1
Baseline WHO stage	Stage I/II	29	275	1	1
	Stage III/IV	32	154	1.80 (1.1–2.98)	1.15 (0.61–2.20)
Past OI	Yes	23	127	1.42 (0.85–2.39)	1.21 (0.61–2.41)
	No	38	302	1	1

*-*p*-value < 0.05 statistically significant

## Discussion

This study investigated the incidence and predictors of virological failure among adult HIV/AIDS patients on first-line ART in northwest Amhara referral hospitals.

The overall incidence rate of virological failure in this follow up was 4.9 events per 1000 PM observations (95%CI: 3.86–6.38). This result was higher than that of a retrospective study done in Adama, Ethiopia [24] 2.1 events per 1000 PM observations. This could be due to differences in a longer duration of ART, and a lower proportion of patients were observed in poor adherence status (11% vs 16%) compared to what is noted in this study. Furthermore, in our study higher proportion (11% vs 8%) of patients were on NVP based first-line ART regimen [35] compared to the report in Adama. Similarly, our study was higher than that done in Thailand [21] with 2.33 events per 1000 PM observations and a retrospective cohort study conducted in Myanmar [29] with 2.7 cases per 1000 PM observations. The discrepancy might be due to the duration of ART and different cut-off points used to define virological failure. Evidence illustrated a short duration of ART increased the risk of virological failure [19, 24]. This is justified by due to early in the initiation of ART, the likelihood of interrupting ARV drugs and developing resistance associated with drug side effects and non-compliance [6, 36] might lead to virological failure. Besides, patients in the Thailand study had good ART adherence (95.7% Vs 84%) compared to our study and this could reduce the burden of other opportunistic infections and prevents viral replication. In Myanmar, virological failure was considered when two consecutive viral load measurements are above 5000 copies/ml, contributing to a low incidence of virological failure. This could increase the incidence of virological failure.

The incidence of virological failure in this study was slightly higher than that of an observational cohort study conducted in South Africa [37] (3.8 events per 1000 PM observations). This can be justified as follows. The study in South Africa was based on a longer duration of ART and used different cut off points to state virological failure. As patients have a longer duration on ART, awareness about the importance of taking ART could increase, and the common, early and severe adverse drug reactions might also decrease [38, 39].

This, in turn, will lead to an increase in the possibility of adherence and boosted immunity which increases viral suppression. In South Africa, treatment failure was declared when the patient viral load on two consecutive measurements was greater than 5000 copies/ml; this might underestimate the incidence rate of virological failure.

The finding of this study was in line with that of a study done in northwestern Uganda [19] with 4.83 events per 1000 PM observations. This similarity could be due to a similar duration of ART and with a similar cut off points of viral load to define virological failure.

Furthermore, this work reported a lower incidence of virological failure than the study conducted somewhere else [20, 40]. The study done in India reported that the overall incidence rate was 8.92 events per 1000 PM observations. This might be due to variations in ART treatment durations, exclusion criteria used and the definition of virological failure. Regarding the exclusion criteria, the study done in India excluded patients with a high baseline CD4 count. CD4 count has an inverse relationship with viral load in that high baseline CD4 count prevents the replication of the virus thereby increasing patient immunity which in turn reduces virological failure [39]. The shorter duration of ART treatment in India and the viral load measurement used to define virological failure on two consecutive samples (above 400 copies/ml) were the reasons for the differences.

Similarly, the result of this study was lower than that of a study done in Jinia, southeast Uganda with the incidence rate of 7.23 events per 1000 PM observations. The higher incidence of virological failure in Uganda could be due to methodological differences (randomized equivalence trial). RCT by itself increases the attrition rate of the patients but the method of analysis used in the Southeast Uganda study was intended to treat. Intent to treat analysis considers all patients assigned at the beginning of an event despite the follow-up time with the first viral load result which is greater than 500 copies/ml. Regarding cut off points of virological failure, in Southeast Uganda, virological failure was defined as viral load above 500 copies/ml, and a high proportion (78% Vs 10.82%) of patients have treated in NVP based regimen [35].

The other possible reason might be the inclusion criteria used. For example, the study participants in Southeast Uganda were WHO stage IV or late-stage III disease or CD4 count below 200 cells/mm^3^.

Also, higher sample size was used in Southeast Uganda compared to this study. When the sample size increases, the probability of getting a high number of events also increases. Thus, all these differences could overestimate the incidence rate of virological failure in southeast Uganda.

According to the Weibull regression model, non-user CPT, fair/poor ART adherence, NVP based first-line regimen, and baseline lower CD4 count (<=200 cells/mm^3^ and 201–350 cells/mm^3^) were significant predictors of virological failure.

This study showed that patients who were on CPT had a lower chance of developing virological failure by 45%. This can be justified by the fact that CPT boosts the immune status of patients in that CPT directly prevents opportunistic infections, and leads to the reduction in the incidence of virological failure associated with different causes. This has been supported by daily co-trim oxazole prophylaxis was associated with reduced morbidity and mortality and had beneficial effects on CD4-cell count and viral load. CPT increases CD4 count and reduces viral loads on ART patients. On the other hand, viral load increases before the introduction of CPT but decreases during taking CPT [34].

Poor adherence was also found to be the other predictor of virological failure. The risk of developing virological failure of patients with poor adherence was 5 times more than t that of patients with good adherence. The result was consistent with those of studies in Thailand [21], Mozambique [41], Rwanda [30], Kenya [42], Harare [33], rural Uganda [23], Tanzania [43], Adama [24], Tigray [17], Dessie [26] and Gondar [25]. It is a common agreement that adherence issues are the most important point for ART users and that is why poor adherence increases the risk of virological failure. Evidence showed that when the adherence level is below 95%, patients are prone to develop drug resistance and low immunity [44], and in poor adherent patients CD4 count significantly decreases and leads to immunological failure [45]. This creates an appropriate condition for viral replication and leads to virological failure.

This study reported that the hazards of developing virological failure among patients who were treated in the NVP based first-line ARV drug regimen were three and half times higher than that of patients who were treated in EFV based regimen. This was supported by studies done in South Africa [22, 27, 37], AIDS relief site countries (Kenya, Nigeria, and Zambia) [31] and Uganda [46]. NVP’s favor for the development of drug resistance and the pill burden associated with concomitant treatments could lead to reduced immunity and decreased adherence [47]. Studies indicated that in resource-limited settings, anti-retroviral drug regimens mostly consisted of non-nucleoid reverse transcriptase inhibitors typically NVP. Hence, 18% of the patients who started in the NVP based first-line ART regimen were prone to treatment failure due to drug toxicity [48]. According to the recommendations of the Ethiopian Federal Ministry of Health in January 2019, NVP based regimen has to be phased out as of September 2019 due to drug-drug interaction, toxicity and lower genetic barrier against ART resistance [35]. This could accelerate viral replication by enhancing immunological and clinical failures and significantly increasing virological failure.

Another important predictor of virological failure was baseline CD4 count in which patients with a CD4 count of (<=200 cells/mm^3^) and (201–350 cells/mm^3^) were nearly four times at higher hazard of developing virological failure compared to patients with more than 500 cells/mm^3^ CD4 count. This result was similar to those of studies done in Myanmar [29], Kenya [42], AIDS relief site countries (Kenya, Nigeria and Zambia) [31], Nigeria [28], Swaziland [49], rural Gabon [50], south Africa [51],Tigray [17], Dessie [26] and Gondar [25]. It is known that viral replication has an inverse relationship with CD4 count, and lower CD4 count increases the risk and occurrence of opportunistic infections and high an attrition rate [52]. Patients with drug resistance or interruption have immunological failure and may reflect viral replication [53]. There appears to be a consistent relationship between current low CD4 count and the hazard of virological failure. Besides, patients with compromised immunity are more susceptible to different opportunistic infections that ultimately lead to increased virological failure [44].

The limitation of this study was based on secondary data, and follow up data (CD4 count and T-staging) was incomplete and didn’t incorporated as a predictors and also didn’t studied behavioral characteristics like smoking, alcohol and psychosocial, emotional factors like stigma, depression and anxiety.

## Conclusion

The incidence rate of virological failure was high among HIV patients on the first line ART at northwest Amhara referral hospitals, Northwest, Ethiopia. Non users of CPT, poor adherence, NVP based regimen and lower CD4count (<=200 cells/mm^3^ and 201–350 cells/mm^3)^ were independent predictors associated with increased risk of virological failure. Patients with this condition need to be carefully observed during the follow up time. Furthermore, since NVP based regimen is associated with virological failure. This is very important to facilitate shifting NVP based regimen to the recommended drug (DTG based).

## Data Availability

Based on reasonable request you can get the data used for the current analysis from the corresponding Author.

## Abbreviations

AHR: Adjusted hazard ratio
AIC: Akaike information criterion)
AIDS: Acquired immune deficiency syndrome
BIC: Bayesian information criterion
CHR: Crude hazard ratio
CPT: Cotrimoxazole preventive therapy
HIV: Human Immune virus
LL: Log-likelihood ratio
NVP: Neverapine
PYFU: person year follow up
